# SEOM Clinical Guideline in ovarian cancer (2016)

**DOI:** 10.1007/s12094-016-1588-8

**Published:** 2016-11-30

**Authors:** A. Santaballa, P. Barretina, A. Casado, Y. García, A. González-Martín, E. Guerra, N. Laínez, J. Martinez, A. Redondo, I. Romero

**Affiliations:** 1 Servicio de Oncología Médica, Hospital Universitari I Politècnic La Fe, Avda de Fernando Abril Martorell, n. 106, 46026 Valencia, Spain; 2 Servicio de Oncología Médica, Institut Català d’Oncologia, Girona, Spain; 3 Servicio de Oncología Médica, Hospital Clínico Universitario, San Carlos, Madrid, Spain; 4 Servicio de Oncología Médica, Corporació Sanitària Parc Taulí, Sabadell, Spain; 5 Servicio de Oncología Médica, MD Anderson Cancer Center, Madrid, Spain; 6 Servicio de Oncología Médica, Hospital Universitario Ramón y Cajal, Madrid, Spain; 7 Servicio de Oncología Médica, Complejo Hospitalario de Navarra, Pamplona, Spain; 8 Servicio de Oncología Médica, Hospital Universitario Virgen de la Arrixaca, Murcia, Spain; 9 Servicio de Oncología Médica, Hospital Universitario La Paz, Madrid, Spain; 10 Servicio de Oncología Médica, Fundación Insituto Valenciano de Oncología, Valencia, Spain

**Keywords:** Ovarian cancer, Treatment guidelines, First line, Recurrent disease

## Abstract

Despite remarkable advances in the knowledge of molecular biology and treatment, ovarian cancer (OC) is the first cause of death due to gynecological cancer and the fifth cause of death for cancer in women in Spain. The aim of this guideline is to summarize the current evidence and to give evidence-based recommendations for clinical practice.

## Introduction

Despite continuous advances in hereditary ovarian cancer (OC) identification to prevent it, surgical efforts in the upper abdomen, new insights in molecular heterogeneity, and new therapies, OC remains the most lethal gynecological cancer [1]. Most patients will present with advanced FIGO stage III or IV disease and around two-thirds will ultimately relapse. In this scenario, the increase in quality of life and survival is based on a multidisciplinary approach. The aim of this guideline is to summarize the current evidence and to give evidence-based recommendations for clinical practice.

## Methodology

SEOM guidelines have been developed with the consensus of ten OC oncologists from the cooperative groups GEICO and SEOM. To assign a level and quality of evidence and a grade of recommendation to the different statements of this treatment guideline, the Infectious Diseases Society of America-US Public Health Service Grading System for Ranking Recommendations in Clinical Guidelines was used (Table 1). The final text has been reviewed and approved by all authors.

**Table 1:** Strength of recommendation and quality of evidence score

Category, grade	Definition
Strength of recommendation	
A	Good evidence to support a recommendation for use
B	Moderate evidence to support a recommendation for use
C	Poor evidence to support a recommendation
D	Moderate evidence to support a recommendation against use
E	Good evidence to support a recommendation against use
Quality of evidence	
I	Evidence from ≥1 properly randomized, controlled trial
II	Evidence from ≥1 well-designed clinical trial, without randomization; from cohort or case-controlled analytic studies (preferably from >1 center); from multiple time series; or from dramatic results from uncontrolled experiments
III	Evidence from opinions of respected authorities, based on clinical experience, descriptive studies, or reports of expert committees

## Pathology and molecular biology

The correct identification of different EOC histologic subtypes [2] is becoming more challenging and of the foremost importance because of its increasing prognostic, therapeutic implications, and increasingly distinct clinical trials. Together with morphology, the widespread use of immunohistochemistry with WT1, p53, NAPSIN A, beta catenin, and progesterone receptor can help refine up to 95% of cases and increase the interobserver agreement [III, A] [3]. This information and its molecular counterpart are useful tools (Table 2).

**Table 2:** Most characteristic IHC staining and relevant molecular features in epithelial ovarian cancer

	IHC				Molecular features						
	Abnormal p53	WT1	NAPSIN A	PR	gBRCA 1/2 (%)	PI3KCA (%)	HER2 (%)	KRAS (%)	BRAF (%)	P53 mut. (%)	ARID1A mut. (%)
HGSC	+	+	–	+	20					96	
**EC**	–	−/+	−/+	+	8						30
**CCC**	–	–	+	−/+	6	33	14	14			46
**LGSC**	–	+	–	+	<10		8	41	6		
**MC**	–	–	–	–	0	5	14	65			

*HGSC* High-grade serous carcinoma, *EC* endometrioid carcinoma, *CCC* clear cell carcinoma, *LGSC* Low-grade serous carcinoma, *MC* mucinous carcinoma, *Abnormal p53* stands for <1 and >70% staining, *WT1* Wilms Tumour 1, *PR* Progesterone Receptor, *gBRCA1/2* germline deleterious mutations, *HER2* amplification, *p53* mut. for mutation

Low-grade serous carcinoma (LGSC) can be differentiated from high-grade serous carcinoma (HGSC) using morphology and p53, and implementing the binary system has been introduced into the new WHO classification. Together with p53 positivity, sometimes, endometrioid histology is classified as HGSC thus amenable to be treated with PARP inhibitors. The new classification further refines borderline histology and the use of SEE-FIM protocol in ovarian cancer can be used to distinguish its origin [4].

## Surgical treatment

Surgery is a mainstay in staging and treatment of OC. Primary surgery must be performed by gynaecologic oncologist surgeons [II, A].

### Early disease (clinical stage I–II)

At diagnosis, 15–20% of woman have FIGO stage I disease. Surgery staging in these patients provides prognostic information and influences advice regarding adjuvant chemotherapy (CT). Surgical staging for OC originally required an exploratory laparotomy to perform the various procedures recommended by FIGO: peritoneal washings, bilateral salpingo-oophorectomy, hysterectomy, multiple peritoneal biopsies, at least infracolic omentectomy, appendectomy in case of mucinous histology, and pelvic and para-aortic lymph node dissection up to the renal veins [5] [II, A].

Laparotomy has been the standard procedure for surgical staging in OC; however, several retrospective series and meta-analysis establish that laparoscopic approach in the early stages has comparable results to laparotomy in terms of the surgical outcomes and oncological safety [6, 7] and could be adequate and feasible for the treatment of early stage OC [II, A].

Lymphadenectomy is recommended in the early stage OC in non-mucinous histological subtypes, as it allows complete staging that provides prognostic information and is associated with greater OS [8] [II, A].

When young women are affected, fertility sparing surgery could be considered in the early stage disease. Patient should be clearly informed about the possible risk of recurrent OC. Patients with stage I with unilateral ovarian involvement and favorable histology (grade 1 or 2 mucinous, serous, endometrioid, or mixed histology) would be amenable to organ preserving surgery but only in combination with complete surgical staging. After fulfilling their wishes of fertility, salpingo-oophorectomy is recommended [III, B].

### Advanced disease (clinical stage III–IV)

In advanced stages, the surgical approach must be an open laparotomy to determine the real extent of the disease, define the stage according to the new FIGO classification (Table 3), and establish surgical techniques to perform. Cytoreduction is associated with increased survival. The volume of residual disease remaining after cytoreductive surgery correlates inversely with survival. Moreover, the main objective of this initial surgery is to obtain an optimal cytoreduction, defined [9] “as the absence of macroscopic residual disease” [II, A]. To achieve this, more complex surgical techniques may be necessary in upper abdomen.

**Table 3:** FIGO classification 2014

Stage I	Tumour confined to ovaries or fallopian tube(s)
IA	Tumour limited to 1 ovary (capsule intact) or fallopian tube; no tumour on ovarian or fallopian tube surface; no malignant cells in the ascites or peritoneal washings
IB	Tumour limited to both ovaries (capsules intact) or fallopian tube; no tumour on ovarian or fallopian tube surface; no malignant cells in the ascites or peritoneal washings
IC	Tumour limited to 1 or both ovaries or fallopian tubes, with any of the following:
IC1	Surgical spill
IC2	Capsule ruptured before surgery or tumour on ovarian or fallopian tube surface
IC3	Malignant cells in the ascites or peritoneal washings
Stage II	Tumour involves 1 or both ovaries or fallopian tubes with pelvic extension (below pelvic brim) or primary peritoneal cancer
IIA	Extension and/or implants on uterus and/or fallopian tubes and/or ovaries
IIB	Extension to other pelvic intraperitoneal tissues
Stage III	Tumour involves 1 or both ovaries or fallopian tubes, or primary peritoneal cancer, with cytologically or histologically confirmed spread to the peritoneum outside the pelvis and/or metastasis to the retroperitoneal lymph nodes
IIIA1	Positive retroperitoneal lymph nodes only (cytologically or histologically proven):
IIIA1(i)	Metastasis up to 10 mm in greatest dimension
IIIA1(ii)	Metastasis more than 10 mm in greatest dimension
IIIA2	Microscopic extrapelvic (above the pelvic brim) peritoneal involvement with or without positive retroperitoneal lymph nodes
IIIB	Macroscopic peritoneal metastasis beyond the pelvis up to 2 cm in greatest dimension, with or without metastasis to the retroperitoneal lymph nodes
IIIC	Macroscopic peritoneal metastasis beyond the pelvic more than 2 cm in greatest dimension, with or without metastasis to the retroperitoneal lymph nodes (includes extension of tumour to capsule of liver and spleen without parenchymal involvement of either organ)
Stage IV	Distant metastasis (excludes peritoneal metastasis)
IVA	Pleural effusion with positive cytology
IVB	Parenchymal metastases and metastases to extra-abdominal organs (including inguinal nodes and lymph nodes outside the abdominal cavity)

Prat J. International Journal of Gynecology and Obstetrics 2014; 124: 1–5

The value of pelvic and para-aortic lymphadenectomy in advanced stages still needs to be confirmed prospectively in an undergoing phase III trial and current data are based on an improvement in OS in a combined analysis of three randomized trials conducted by the AGO-OVAR group [10]. Therefore, a pelvic and para-aortic lymphadenectomy is recommended [II, A].

Contraindications for this upfront maximal debulking surgery have been defined as: poor performance status, mesentery root involvement, extra-abdominal visceral disease, multiple intraparenchymal liver metastases, or intestinal massive-serosal carcinomatosis. [II, A].

### Recurrent disease

The benefit of secondary cytoreductive surgery is unclear. The DESKTOP I trial found that surgery with no residual disease was associated with improved survival and identified good performance status [11], no residual tumour after first surgery, and absence of ascites, as predictive factors for complete resection. In the DESKTOP II trial, the predictive value of these factors was validated in patients with disease-free interval ≥6 months and DESKTOP III is currently evaluating it prospectively.

Therefore, surgery at relapse is not a standard treatment, but could be considered in patients with DFI > 6 months, no residual disease after first surgery, good PS, and absence of ascites [II, B].

The use of HIPEC (Hyperthermic Intraperitoneal Chemotherapy) in OC relapses following surgical cytoreduction has no role in OC. There is no evidence from prospective randomized studies and the published studies are very heterogeneous and a pooled analysis has not shown any advantage [12]. HIPEC should not be recommended as treatment after secondary cytoreductive surgery in OC relapse.

## Neoadjuvant chemotherapy

Two phase III randomized studies have reported results in this setting. The EORTC-55971 and the CHORUS trial showed that in women with stage IIIC or IV OC, primary debulking surgery followed by at least six cycles of platinum-based CT or three cycles of platinum-based neoadjuvant CT (NAC), followed by interval debulking surgery, and then at least three more cycles of platinum-based CT, achieved the same OS [13, 14]. However, some concerns have arisen regarding the quality of the surgery performed and the use of NAC in candidates for optimal upfront debulking surgery. Only the subset of patients with upfront surgery and no residual disease seemed to achieve higher survival rates in a subgroup analysis.

NAC should be reserved for those patients who cannot tolerate PDS and/or for whom optimal cytoreduction is not feasible after an adequate evaluation performed by an expert surgical team [I, B]. Otherwise, PDS followed by adjuvant platinum and taxane combination is the recommended standard treatment [I, A].

## Initial systemic therapy

### Early stages

Adjuvant platinum-based CT after surgery is indicated in high-risk early stages (IA and IB Grade 3, clear cell tumours, and any grade of stages IC and IIA) [15] [I, A]. Only low-risk patients (stages IA/B Grade I) with complete and comprehensive surgical staging require observation exclusively. The recommended regimen consists of at least three cycles of paclitaxel-carboplatin [I, A], although six cycles should be recommended in high-grade serous histology [II, B].

### Advanced stages

Different options of first-line CT are available for advanced stage (III–IV) OC patients: conventional CT, dose-dense, intraperitoneal, or conventional CT combined with antiangiogenics. In Table 4, the different strategies in this setting are summarized.

**Table 4:** Systemic therapy options after upfront surgery or interval debulking surgery (preferred options are selected based on evidence)

	IV chemotherapy without bevacizumab	IV chemotherapy with bevacizumab	IP chemotherapy
Upfront surgery			
Stage III with RD ≥1 cm Stage IV	Option	Preferred option	Not indicated
Stage III with RD <1 cm	Option	Option	Preferred option*
Stage III without RD	Option	Option	Preferred option*
Interval debulking surgery			
Stage III with RD ≥1 cm Stage IV	Option	Option	Not indicated
Stage III with RD <1 cm	Option	Option	Option
Stage III without RD	Option	Option	Option

* In fit patients

#### Conventional chemotherapy

Standard post-operative treatment in advanced stages after complete surgical staging consists of a combination of carboplatin (AUC 5–6) and paclitaxel (175 mg/m^2^) every 3 weeks for 6 cycles [I,A]. Alternative CT regimens, intraperitoneal regimens, or the addition of bevacizumab will be discussed below. For patients not eligible to receive a taxane (specifically paclitaxel), the combination of carboplatin and pegylated liposomal doxorubicin (PLD) is recommended [16] [I, B].

#### Dose-dense therapy

A dose-dense regimen was superior to 3-weekly carboplatin plus paclitaxel in a large, randomized Japanese trial (JGOG 3062), with increased PFS and OS, but worse toxicity profile, mainly hematological and neuropathy [17]. However, the results of the MITO 7 trial that compared a weekly paclitaxel and carboplatin schedule with 3-weekly carboplatin and paclitaxel, and the GOG 262 trial that compared, the weekly paclitaxel and carboplatin schedule with the 3-weekly schedule regardless of bevacizumab exposure have not confirmed the benefit of the dose-dense regimens in Caucasian population. For this reason, dose-dense therapy cannot be considered the standard of care in first-line setting [I, B].

#### Intraperitoneal chemotherapy

Three large randomized studies (GOG 104, GOG 114, and GOG 172) and one meta-analysis have found clinically significant improvements in PFS and OS when part of the CT is administered directly in the peritoneal cavity after upfront surgery. GOG 172 showed an improvement of median OS from 49.5 to 66.9 months, favorable to the IP CT arm, but with a significant toxicity [18]. A recent update of GOG 114 and GOG 172 studies concluded that OS advantage of intraperitoneal (IP) CT extends beyond 10 years and improves with increasing number of IP cycles [19]. GEICO group published an outpatient modified intraperitoneal regimen that resulted in a lesser toxicity and a greater rate of treatment completion than previously reported [20].

To conclude, IP CT is shown to be superior to IV CT after primary debulking surgery and is another standard option in the management of selected patients with stage III with optimal surgery or residual tumour ≤1 cm [I, A]. The role of IP CT after interval debulking surgery is controversial, although it could be an option for some patients [I, B].

#### Antiangiogenic therapy

Phase III data are currently available in front-line therapy on Bevacizumab, Pazopanib, and Nintedanib.

Two large randomized studies (GOG 218 and ICON 7) have reported that bevacizumab added to the initial CT followed by maintenance period with bevacizumab improves PFS in comparison with standard CT alone in patients with FIGO III and IV OC. The improvement in PFS was 3.8 months (HR = 0.72) in GOG trial and 1.5 months (HR = 0.81) in ICON 7. According to a meta-analysis, the benefit in OS of 4.8 months is observed in patients with either stage III and residual disease >1 cm, or stage IV disease [21, 22].

Bevacizumab added to the initial CT followed by a maintenance period of bevacizumab should be included for patients who, following standard surgery, have macroscopic residual disease [I, A].

Pazopanib and Nintedanib are not approved for OC treatment.

## Treatment of recurrent disease

### Factors to consider when selecting therapy in recurrent ovarian carcinoma

Approximately, 50–90% of patients with advanced OC will have a relapse in the first 5 years after the diagnosis depending on the initial FIGO stage at presentation, use of neoadjuvant CT, and residual disease after upfront cytoreductive surgery. Treatment of patients with recurrent disease is a great challenge due to the heterogeneity of disease and clinical situations. We need to consider many different factors for selecting the different therapy of the relapse (Table 5).

**Table 5:** Treatment options in relapsed ovarian cancer

TFIp > 6 months		TFIp < 6 months
BRCA-mutated	Non-BRCA-mutated	
Non-previous bevacizumab		
Platinum combination and maintenance with olaparib (IA) Carbo-gem and bevacizumab (IA) Platinum combination (IA) PLD + trabectedin* (IB)	Carbo-gem and bevacizumab (IA) Platinum combination (IA) PLD + trabectedin* (IB)	Single-agent (weekly paclitaxel, PLD or topotecan) + bevacizumab (IA) Single-agent (weekly paclitaxel, PLD or topotecan, gemcitabine) (IA)
Previous bevacizumab		
Platinum combination and maintenance with olaparib (IA) Platinum combination (IA) PLD + trabectedin* (IB)	Platinum combination (IA) PLD + trabectedin* (IB)	Single-agent (weekly paclitaxel, PLD or topotecan, and gemcitabine) (IA)

* If platinum is not an option

Factors depending on the tumour

- The site of disease and extension to consider surgical options.
- Histological subtype.
- BRCA1/2 status to identify candidates to olaparib.

Factors depending on the patient

- Treatment-free interval: Platinum-free interval (TFIp) has been considered classically a predictive factor of response to platinum-rechallenge. Patients have been divided in platinum-resistant, partially platinum-sensitive, and platinum-sensitive according to the TFIp (less than 6, 6–12, or >12 months).
- Type of previous therapy: It should be considered the previous use of cytotoxic agents and response obtained, as well as the previous use of targeted agents like antiangiogenic therapy or PARP inhibitors.
- Residual toxicity after the previous lines.
- Co-morbidities of the patient and special geriatric population.
- Preference and expectations of the patient.

### Relapse with platinum-free interval >6 months

A platinum-based combination is associated with a longer PFS and OS in comparison to single-agent platinum. There is no combination that can be considered superior in terms of efficacy; the schedule selection should be based on the toxicity profile [18].

A randomized phase III trial of bevacizumab combined with carboplatin-gemcitabine, in patients in first relapse who have not been treated with antiangiogenic therapy, has shown a benefit in RR and PFS [23]. The combination of bevacizumab with carboplatin and paclitaxel in this setting has also shown improvement in PFS [24].

In patients with HGSC OC platinum-sensitive relapse and *BRCA1/2* mutation who respond to platinum, the maintenance treatment with olaparib improves PFS with a HR of 0.18 and an increment in median PFS from 4.3 to 11.2 months, but the trial was underpowered for OS [25].

Patients with TFIp of 6–12 months have lower response rates to platinum and different strategies beyond carboplatin-based regimens are under investigation. A subgroup analysis of a randomized trial comparing trabectedin and pegylated liposomal doxorubicin (PLD) with PLD showed that those patients with TFIp of 6–12 months treated with the non-platinum combination and a platinum-based therapy at progression obtained a benefit in OS [26]. However, a randomized clinical trial has shown that the use of non-platinum single agent followed by platinum in patients with relapsed ovarian cancer and a PFI of 6–12 months was inferior to platinum-based combination [27].

In patients with TFIp >6 months, the standard treatment is a platinum combination [I, A], with the consideration of adding bevacizumab in first relapse if the patients have not been treated with bevacizumab in first line [I, A]. In BRCA-mutated patients who respond to platinum, maintenance with olaparib must be considered [IA]. In patients with TFIp 6–12 months, a platinum combination [I, A] or trabectedin-PLD (I, B) could be considered.

### Relapse with platinum-free interval <6 months

Patients with TFIp <6 months have poor prognosis. There is no I level evidence of active treatment versus best supportive care in this clinical setting. Yet, it is known that patients progressing on two consecutive lines of treatment should be considered for best supportive care or clinical trials depending on their performance status [18].

These patients should be treated with sequential single-agent CT to improve symptom control and quality of life. Palliative chemotherapies accepted are PLD, weekly paclitaxel, topotecan, and gemcitabine [18]. They have shown activity in several phase III trials with response rates of less than 20%, a median PFS of 3–4 months, and a median OS of 9–12 months. None of them has proven to be superior in terms of RR, PFS, and OS. Clinician must choose wisely based on the above-mentioned criteria.

For platinum-resistant patients who have not received neither more than two previous lines nor prior bevacizumab, the addition of the latter to weekly paclitaxel, PLD, or topotecan has shown to improve PFS (3.4–6.7 months) and OS (13.3 vs 16.7 months) [28]. This combination therapy also significantly improved symptoms with a significantly higher proportion of patients achieving the predefined 15% improvement in abdominal/GI symptoms.

In platinum-resistant OC patients, single-drug therapy or a combination with bevacizumab in case they have not received this drug previously is recommended [I, A].
